# Supplementary material for: The rapid and highly parallel identification of antibodies with defined biological activities by SLISY
Source: Nat Commun. 2023 Jan 3;14:17. doi: 10.1038/s41467-022-35668-6 (PMC9808734; doi:10.1038/s41467-022-35668-6)
Supplement: Supplementary file 1 — Supplementary Information [file 41467_2022_35668_MOESM1_ESM.pdf]

Supplementary Information

The rapid and highly parallel identification of antibodies with defined biological activities by SLISY

Steve Lu[1,2], Austin K. Mattox[1,2], P. Aitana Azurmendi[3], Ilias Christodoulou[4], Katharine M. Wright[3], Maria Popoli[1,2], Zan Chen[3], Surojit Sur[1,2], Yana Li[3], Challice L. Bonifant[4], Chetan Bettegowda[1,2], Nickolas Papadopoulos[1,2], Shibin Zhou[1,2], Sandra B. Gabelli[3,4], Bert Vogelstein[1,2], and Kenneth W. Kinzler[1,2,*]

*Corresponding author: kinzlke@jhmi.edu

Contents

Supplementary Figure 1. Primer design for long-read sequencing.

Supplementary Figure 2. Distribution of SARS-CoV-2 specific clones after biopanning.

Supplementary Figure 3. Selected SARS-CoV-2 mAbs.

Supplementary Figure 4. Epitope binning of SARS-CoV-2 antibodies.

Supplementary Figure 5. Assessing clones across multiple SARS-CoV-2 variants using SLISY.

Supplementary Figure 6. Gating for HLA-A3 expressing cells.

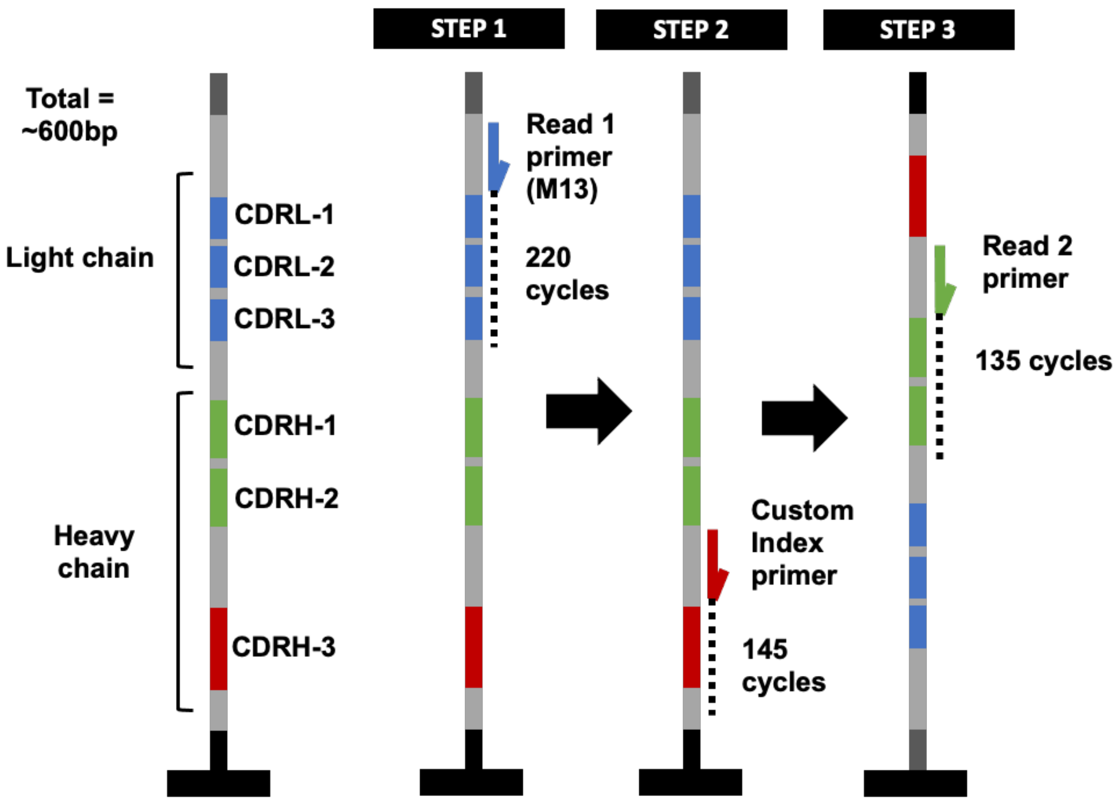

Supplementary Figure 1. Primer design for long-read sequencing. Initial amplification of template was done using forward and reverse primers flanking the entire scFv sequence. Amplified template was then used for long-read sequencing on an Illumina Sequencer. Read 1 primer was used to sequence CDRL-1, -2, and -3 regions as well as the molecular barcodes. A custom index primer was used to sequence the highly diverse CDRH-3 as well as the sample index. A custom read 2 primer was used to sequence the CDRH-1 and CDRH-2 regions. CDRL - complementarity determining region of light chain; CDRH - complementarity determining region of heavy chain.

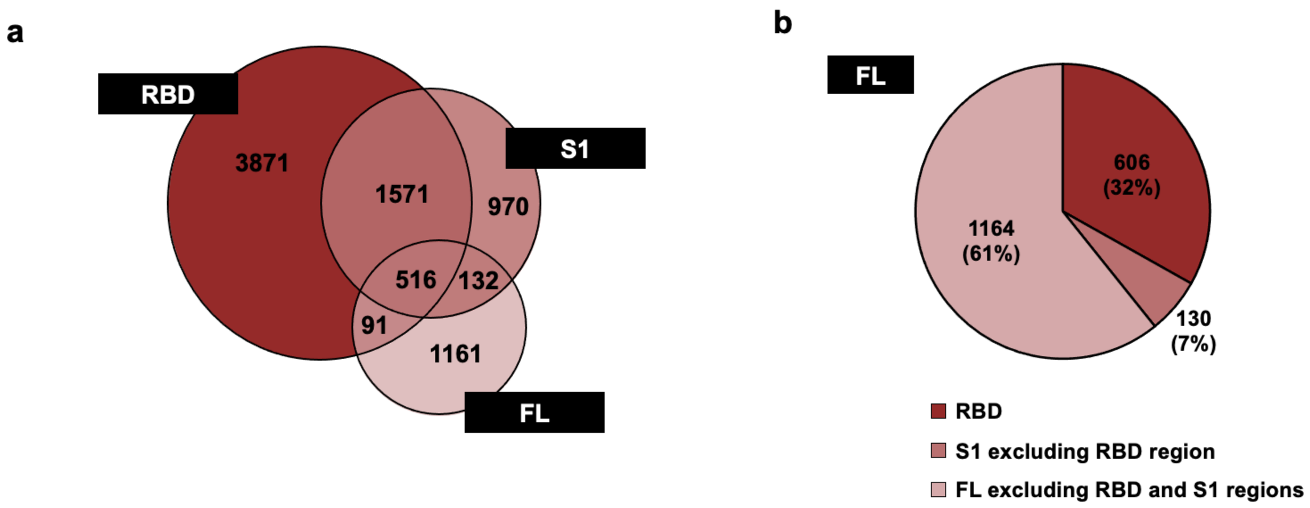

Supplementary Figure 2. Distribution of SARS-CoV-2 specific clones after biopanning.

a. Overlap of SARS-CoV-2 candidate clones from the biopannings of RBD, S1, and FL spike protein. Binding of clones along the spike protein after four rounds of biopanning was detected by SLISY. Only clones that had SBR greater than 10 were considered. 516 unique clones were independently selected and identified in all three biopannings. b. Localization of clones selected around FL spike protein. To determine the relative regions where the clones bound on SARS-CoV-2 spike protein, the polyclonal phage from four rounds of FL biopanning was applied to the RBD, S1, and FL spike proteins. Independent SBRs determined using SLISY. Of the clones that were selected using the FL spike protein, 32% bound to the RBD region. Source data are provided as a Source Data file.

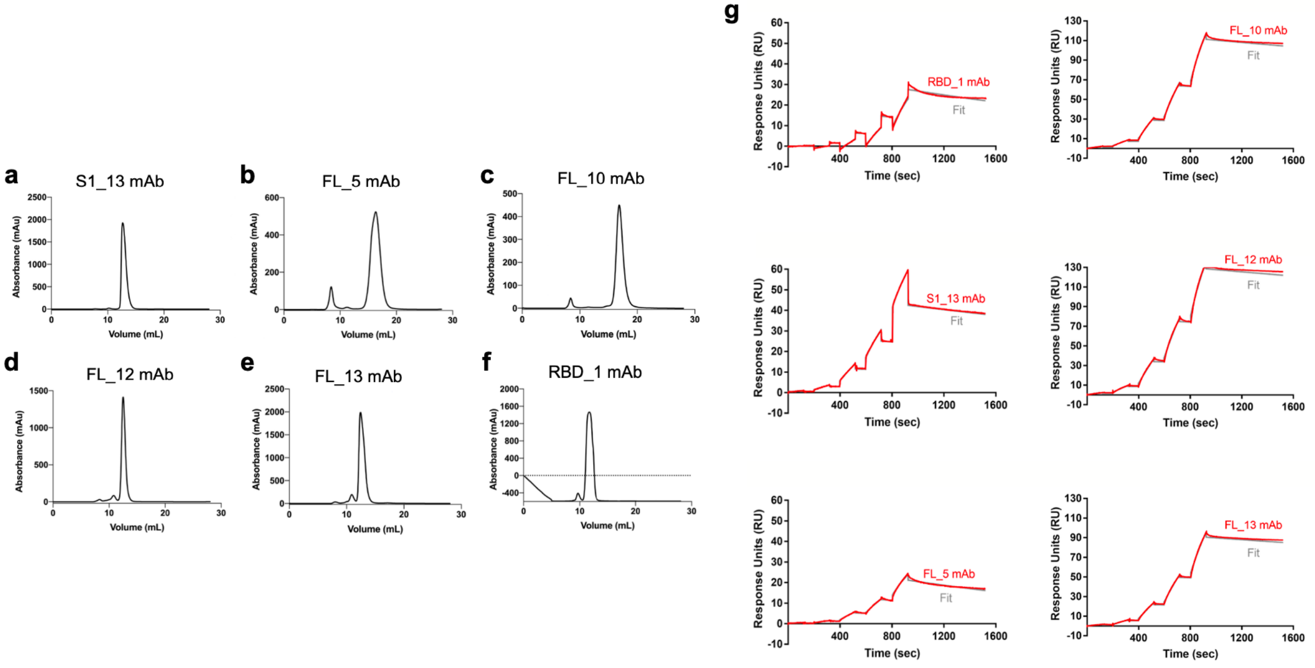

Supplementary Figure 3. Selected SARS-CoV-2 mAbs. a-f. Gel filtration chromatograms of S1_13, FL_5, FL_10, FL_12, FL_13, and RBD_1 mAbs, respectively; mAbs eluted at ~12 mL or ~18 mL with a 20 mM Na Phosphate, pH 7.2, 150 mM NaCl buffer. Chromatograms are representative of three replicates. g. Affinity of SLISY selected-mAbs to SARS-CoV-2 protein as measured by SPR. RBD_1 mAb binding to SARS-CoV-2 FL spike protein was assessed with single-cycle kinetics using SPR (red line). The RBD_1 mAb was attached to a protein A chip and SARS-CoV-2 FL spike protein was loaded at increasing concentrations (5 nM, 20 nM, 80 nM, 160 nM, 320 nM). RBD_1 mAb bound to the SARS-CoV-2 FL spike protein with a K_D of 44.2 nM when fit to one-to-one binding kinetics (grey line). The blank- and reference-subtracted binding curve is shown. Other studies carried out in the same way as RBD_1 but for the following selected mAbs: FL_10 (K_D of 7.5 nM), S1_13 (K_D of 11.7 nM), FL_12 (K_D of 6.0 nM), FL_5 (K_D of 39.7 nM), FL_13 (K_D of 9.5 nM) respectively.

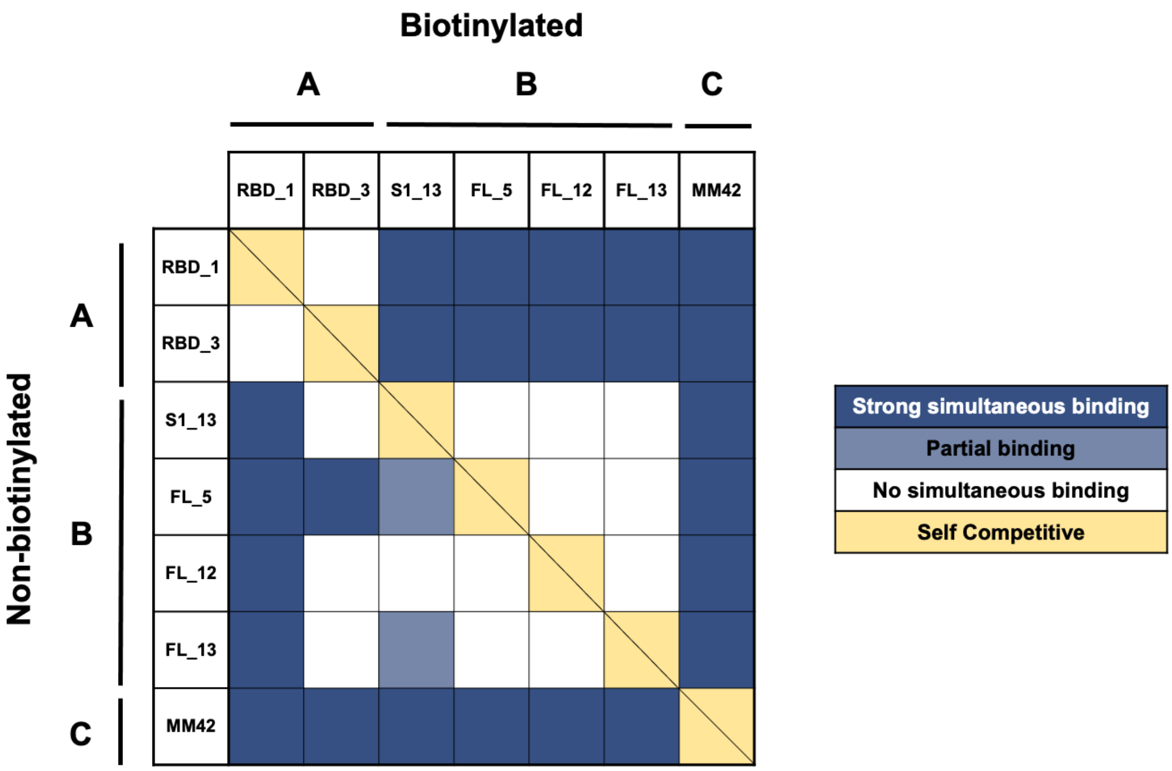

Supplementary Figure 4. Epitope binning of SARS-CoV-2 antibodies. The column indicates the biotinylated mAbs, and the row indicates the competing non-biotinylated mAbs. An indirect ELISA format was used to identify whether isolated neutralizing antibody clones competed for the same antigenic epitope on the SARS-CoV-2 RBD polypeptide. Biotinylated antibody is mixed with each free antibody with the pre-titrated concentration through a volume ratio of 1:1 and applied to an indirect ELISA format to analyze competition within each pair. Inhibition of 70% or greater indicates strong simultaneous binding between the two antibodies likely due to same overlapping epitopes (dark blue squares). Inhibition of 50-70% indicates partial binding (light blue squares), while inhibition less than 50% suggests no simultaneous binding (white squares). Self-competition of an antibody against itself are represented by yellow squares. Source data are provided as a Source Data file.

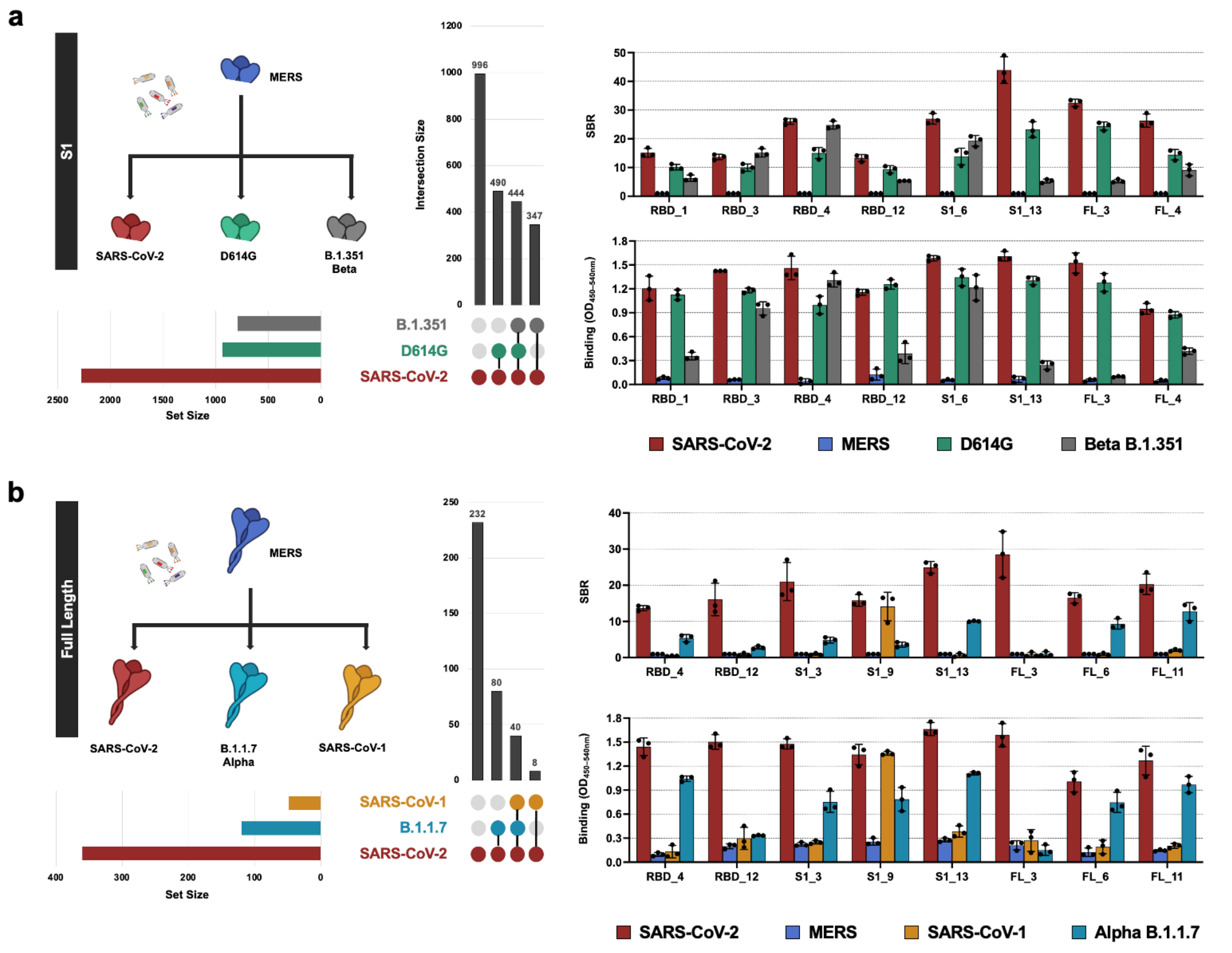

Supplementary Figure 5. Assessing clones across multiple SARS-CoV-2 variants using SLISY. a. S1 variants. b. FL variants. UpSet plots include upright bars that represent the total number of cross-reactive clones in each intersection. Filled circles below bar represent the variants that represent the intersecting set. Horizontal bars represent the total number of clones that had a SBR of ten and above for each of the proteins. Error bars represent standard deviation of means (n=3). Created with BioRender.com. Source data are provided as a Source Data file.

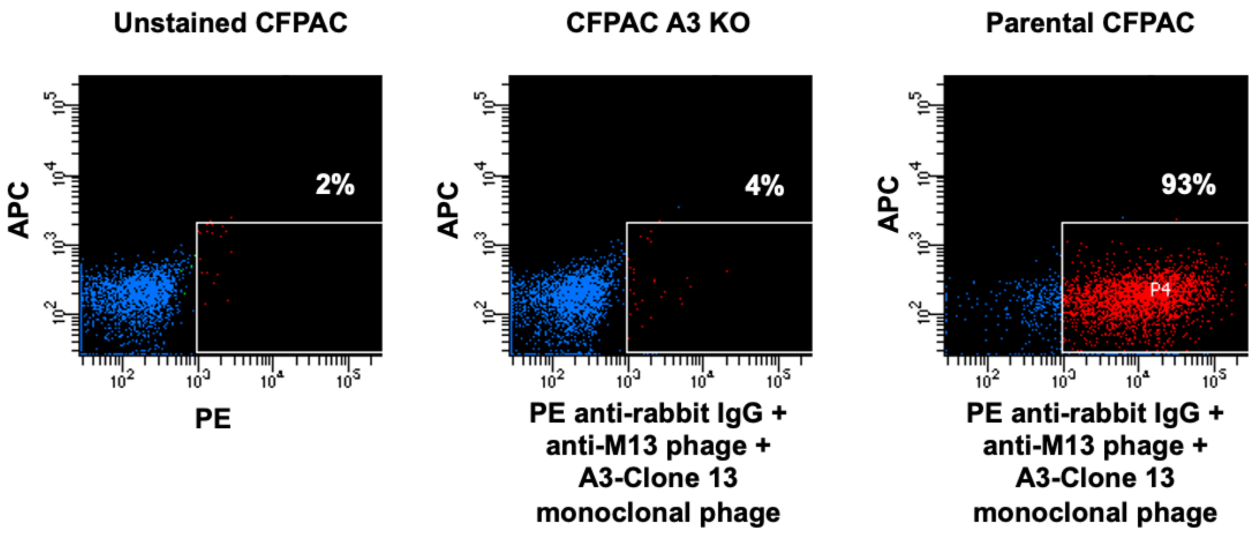

Supplementary Figure 6. Gating for HLA-A3 expressing cells. Representative dot plots of CFPAC parental and A3 KO cell lines. The parental cell line has high HLA-A3 expression that is confirmed when stained with validated HLA-A3 specific monoclonal phage, A3-Clone 13. The A3 KO cell line does not exhibit any staining with A3-Cllone 13 and has similar PE signal to unstained cells. For gating, any PE signal higher than the background signal of unstained CFPAC cells was considered positive.
