# Supplementary material for: The rapid and highly parallel identification of antibodies with defined biological activities by SLISY
Source: Nat Commun. 2023 Jan 3;14:17. doi: 10.1038/s41467-022-35668-6 (PMC9808734; doi:10.1038/s41467-022-35668-6)
Supplement: Supplementary file 3 — Description of Supporting Information [file 41467_2022_35668_MOESM3_ESM.pdf]

Description of Supplementary Data

Supplementary Data 1. Selected HLA A3 clones from cell-based panning

Supplementary Data 2. Amino acid sequence of HLA A3 scFvs tested

Supplementary Data 3. Fraction of selected pools corresponding to top 100 scFvs

Supplementary Data 4. Physical characteristics of potent neutralizing mAbs

Supplementary Data 5. Specificity of full-length SARS-CoV-2 antibody clones for multiple variants

Supplementary Data 6. Neutralization potential of full-length SARS-CoV-2 antibody clones for multiple variants

Supplementary Data 7. Binding activity of Beta, Gamma and Delta selected scFvs

Supplementary Data 8. Neutralization activity of Beta, Gamma and Delta selected scFvs

Supplementary Data 9. Coronavirus antigens

Supplementary Data 10. Long-read sequencing primers
