# Supplementary material for: The rapid and highly parallel identification of antibodies with defined biological activities by SLISY
Source: Nat Commun. 2023 Jan 3;14:17. doi: 10.1038/s41467-022-35668-6 (PMC9808734; doi:10.1038/s41467-022-35668-6)
Supplement: Supplementary file 14 — Reporting Summary [file 41467_2022_35668_MOESM14_ESM.pdf]

nature portfolio

Corresponding author(s): Kenneth W. Kinzler

Last updated by author(s): Jan 31, 2022

Reporting Summary

Nature Portfolio wishes to improve the reproducibility of the work that we publish. This form provides structure for consistency and transparency in reporting. For further information on Nature Portfolio policies, see our Editorial Policies and the Editorial Policy Checklist.

Statistics

For all statistical analyses, confirm that the following items are present in the figure legend, table legend, main text, or Methods section.

n/a	Confirmed	
☐	☒	The exact sample size (*n*) for each experimental group/condition, given as a discrete number and unit of measurement
☐	☒	A statement on whether measurements were taken from distinct samples or whether the same sample was measured repeatedly
☒	☐	The statistical test(s) used AND whether they are one- or two-sided<br>*Only common tests should be described solely by name; describe more complex techniques in the Methods section.*
☒	☐	A description of all covariates tested
☒	☐	A description of any assumptions or corrections, such as tests of normality and adjustment for multiple comparisons
☐	☒	A full description of the statistical parameters including central tendency (e.g. means) or other basic estimates (e.g. regression coefficient) AND variation (e.g. standard deviation) or associated estimates of uncertainty (e.g. confidence intervals)
☒	☐	For null hypothesis testing, the test statistic (e.g. *F*, *t*, *r*) with confidence intervals, effect sizes, degrees of freedom and *P* value noted<br>*Give P values as exact values whenever suitable.*
☒	☐	For Bayesian analysis, information on the choice of priors and Markov chain Monte Carlo settings
☒	☐	For hierarchical and complex designs, identification of the appropriate level for tests and full reporting of outcomes
☒	☐	Estimates of effect sizes (e.g. Cohen's *d*, Pearson's *r*), indicating how they were calculated

Our web collection on statistics for biologists contains articles on many of the points above.

Software and code

Policy information about availability of computer code

Data collection	Illumina MiSeq and HiSeq
Data analysis	The code used in the manuscript to process sequences and translate CDRH3 regions is a custom python software available at Zenodo (https://doi.org/10.5281/zenodo.7154344). The analysis of SLISY by SQL databases (MSSQL) is available from the authors upon request from Kenneth Kinzler (kinzlke@jhmi.edu) within a one week time frame and is limited to non-commercial and research use per Johns Hopkins legal requirements unless explicitly granted by institution.

For manuscripts utilizing custom algorithms or software that are central to the research but not yet described in published literature, software must be made available to editors and reviewers. We strongly encourage code deposition in a community repository (e.g. GitHub). See the Nature Portfolio guidelines for submitting code & software for further information.

Data

Policy information about availability of data

All manuscripts must include a data availability statement. This statement should provide the following information, where applicable:

- Accession codes, unique identifiers, or web links for publicly available datasets
- A description of any restrictions on data availability
- For clinical datasets or third party data, please ensure that the statement adheres to our policy

The data for this study have been deposited in the European Nucleotide Archive (ENA) at EMBL-EBI under accession number PRJEB58033 (https://www.ebi.ac.uk/ena/browser/view/PRJEB58033) and is limited to non-commercial and research use per Johns Hopkins legal requirements unless explicitly granted by institution. There is a sample data set of a MiSeq run of a SLISY experiment for testing that is also available at Zenodo (https://doi.org/10.5281/zenodo.7154344).

Field-specific reporting

Please select the one below that is the best fit for your research. If you are not sure, read the appropriate sections before making your selection.

- [x] Life sciences
- [ ] Behavioural & social sciences
- [ ] Ecological, evolutionary & environmental sciences

For a reference copy of the document with all sections, see nature.com/documents/nr-reporting-summary-flat.pdf

Life sciences study design

All studies must disclose on these points even when the disclosure is negative.

Sample size	The sample size for SLISY was limited and based on the average number of reads available on a MiSeq run (12-20 million reads).
Data exclusions	No data were excluded from the analysis.
Replication	To replicate the sequencing data from SLISY and the functional data by ELISA, all experiments had three replicates so that a standard deviation of the mean could be calculated. All replicates were successful and included.
Randomization	The experiments were performed with randomization meaning that all clones from a pooled sample were applied to the target (spike protein) and allowed to bind.
Blinding	It is blinded in that all clones from a pooled sample were applied to the target (spike protein) and allowed to bind in the fraction that they are enriched in.

Reporting for specific materials, systems and methods

We require information from authors about some types of materials, experimental systems and methods used in many studies. Here, indicate whether each material, system or method listed is relevant to your study. If you are not sure if a list item applies to your research, read the appropriate section before selecting a response.

Materials & experimental systems

n/a	Involved in the study	
[ ]	[x]	Antibodies
[ ]	[x]	Eukaryotic cell lines
[x]	[ ]	Palaeontology and archaeology
[x]	[ ]	Animals and other organisms
[x]	[ ]	Human research participants
[x]	[ ]	Clinical data
[x]	[ ]	Dual use research of concern

Methods

n/a	Involved in the study	
[x]	[ ]	ChIP-seq
[ ]	[x]	Flow cytometry
[x]	[ ]	MRI-based neuroimaging

Antibodies

Antibodies used

1. SARS-CoV-2 spike mouse Mab Monoclonal Mouse IgG1 Clone #42 (40591-MM42) (Sino Biological, Wayne, PA) at 0.1, 1, and 10 µg/mL
2. SARS-CoV-2 spike mouse Mab Monoclonal Mouse IgG2b Clone #57 (40592-MM57) (Sino Biological, Wayne, PA) at 0.1, 1, and 10 µg/mL
3. Rabbit anti-M13 polyclonal Ab (Novus Biologicals, Littleton, CO) at 1:100 for flow cytometry and 1:5000 for ELISA
4. PE Donkey anti-rabbit IgG antibody Clone Poly4064 (Biolegend, San Diego, CA) at 1:100 for flow cytometry
5. Goat anti-rabbit IgG (H+L) antibody HRP Polyclonal (Thermo Fisher, A27036) at 1:10000 for ELISA
6. Goat anti-human IgG Fc-HRP Ab Polyclonal (Abcam, ab98624) at 1:10000 for ELISA

Validation

1. Manufacturer's website: "This antibody was produced from a hybridoma resulting from the fusion of a mouse myeloma with B cells obtained from a mouse immunized with purified, Recombinant SARS-CoV-2 (2019-nCoV) Spike S1-mFc Protein (Catalog#40591-V05H1; YP_009724390.1; Val16-Arg685). The IgG fraction of the cell culture supernatant was purified by Protein A affinity chromatography."
2. Manufacturer's website: "This antibody was produced from a hybridoma resulting from the fusion of a mouse myeloma with B cells obtained from a mouse immunized with purified, Recombinant SARS-CoV-2 (2019-nCoV) Spike RBD-mFc Protein (Catalog#40592-V05H; YP_009724390.1; Arg319-Phe541). The IgG fraction of the cell culture supernatant was purified by Protein A affinity chromatography."
3. Manufacturer's website: "May be used as a reagent in "phage ELISA" offering sensitive and specific activity for detection of recombinant phages."
4. Manufacturer's website: "Each lot of this antibody is quality control tested by immunofluorescent staining with flow cytometric analysis. For flow cytometric staining, the suggested use of this reagent is ≤0.125 µg per million cells in 100 µl volume. It is

recommended that the reagent be titrated for optimal performance for each application."
5. Manufacturer's website: "The sensitivity and specificity of each lot is confirmed using ELISA. Minimal cross-reactivity with mouse, rat, human, bovine, guinea pig and donkey IgG is observed."
6. Manufacturer's website: "Our Abpromise guarantee covers the use of ab98624 in the following tested applications: ELISA"

Eukaryotic cell lines

Policy information about cell lines

Cell line source(s)	Parental CFPAC cell line and HEK-293T cell line purchased from ATCC (CRL-1918)
Authentication	Authentication provided by ATCC using STR profiling
Mycoplasma contamination	Cells were tested negative regularly for Mycoplasma contamination
Commonly misidentified lines (See ICLAC register)	No commonly misidentified lines were used.

Flow Cytometry

Plots

Confirm that:

- [x] The axis labels state the marker and fluorochrome used (e.g. CD4-FITC).
- [x] The axis scales are clearly visible. Include numbers along axes only for bottom left plot of group (a 'group' is an analysis of identical markers).
- [x] All plots are contour plots with outliers or pseudocolor plots.
- [x] A numerical value for number of cells or percentage (with statistics) is provided.

Methodology

Sample preparation	CFPAC parental and HLA-A3 KO lines were harvested, washed with PBS, and resuspended in ice-cold flow cytometry staining buffer (PBS, 0.5% BSA, 2 mM, EDTA, 0.1% sodium azide) at a concentration of 10x106 cells/mL. Next, 10 µL of precipitated phage (1013 titer) was applied to 100 µL of both cells and incubated on ice for 15 min. After washing 3X with staining buffer, cell pellets were resuspended to same concentration (10x106 cells/mL) and stained with 1 µL of rabbit anti-M13 polyclonal antibody (Novus biologicals, Littleton, CO) on ice for 15 min. After washing 3X again, cell pellets were resuspended and stained with 1 µL of PE donkey anti-rabbit IgG antibody (Biolegend, San Diego, CA) for 15 min on ice. Cells were analyzed after a final wash 3X.
Instrument	LSRII
Software	BD FACSDiva
Cell population abundance	100% purity because cell lines
Gating strategy	The gating strategy used autofluorescence from unstained parental cells as negative. A gate was drawn at the end of the parental cells. Any signal above that was considered positive.

- [x] Tick this box to confirm that a figure exemplifying the gating strategy is provided in the Supplementary Information.
